# 1264. Rewrite The SAARs; Experience Implementing Optimized SAAR/AU Dashboards Across a 10 Hospital Health-System

**DOI:** 10.1093/ofid/ofad500.1104

**Published:** 2023-11-27

**Authors:** Hunter O Rondeau, Kimberly Boeser, Kristi Killelea, Meredith B Oliver

**Affiliations:** SSM Health, Bridgeton, Missouri; M Health Fairview - University of Minnesota Medical Center, Minneapolis, Minnesota; M Health Fairview - University of Minnesota Medical Center, Minneapolis, Minnesota; M Health Fairview Masonic Children’s Hospital, Minneapolis, Minnesota

## Abstract

**Background:**

Standardized Antimicrobial Administration Ratios or SAARs are representations of antimicrobial use data and were first provided to hospitals voluntarily participating in the National Health Safety Network Antimicrobial Use (NHSN AU) option in 2015. Submission to the AU module will be mandatory per CMS effective 2024. AU module data is reported back as a SAAR report, with the goal of facilitating risk-adjusted inter- and intra-facility antimicrobial use benchmarking and monitoring antimicrobial use trends over time at the local and national levels. Our health-system implemented optimized SAAR dashboard at its largest hospital and are now expanding

Cumulative percentage of facilities reporting at least one month of data to NHSN’s AU Option

**Figure:**
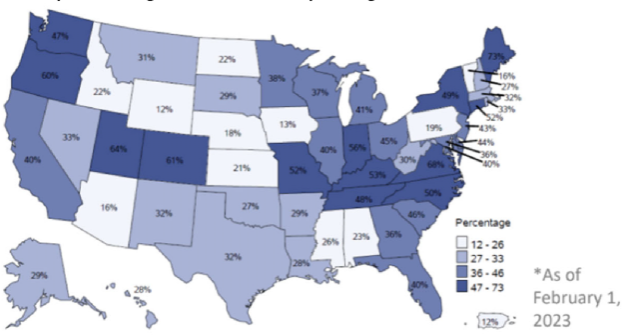

**Methods:**

To optimize our use of this module, we created dashboards for adult and pediatric SAAR antimicrobial categories and consolidated representations of antimicrobials of the corresponding categories and SAAR-reporting locations. Optimization and validation of these dashboards took place at our largest of ten hospitals in the health-system and required collaboration between informatics specialists, infection prevention and antimicrobial stewardship clinicians. To help facilitate implementation, a guidance document was created. Collaboration with informatics specialists, infection prevention and ASP clinicians will occur as these optimized dashboards are adapted to the individual site’s needs, as spelled out in the guidance document.

Optimized Default Dashboard Layout

**Figure:**
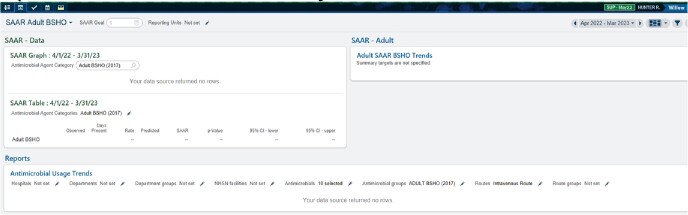

Optimized Default Dashboard Layout for Antimicrobial Category BSHO

**Results:**

Based on the experience of the pilot implementation at the largest of the ten hospitals, the remaining nine hospital’s site, antimicrobial stewardship leads are implementing these optimized dashboards into their antimicrobial stewardship program.

Optimized Dashboard for BSHO

**Figure:**
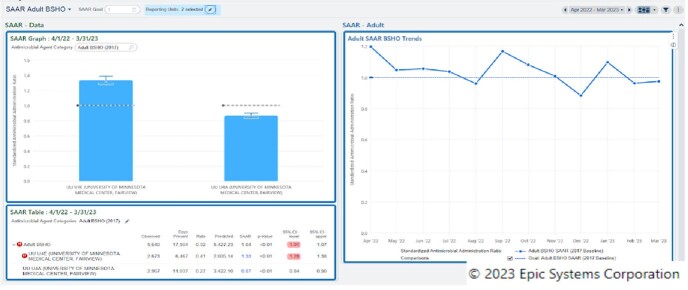

Pictured are two units classified as the same NHSN location type, visualized on the BSHO dashboard.

Optimized Dashboard With Antimicrobial and NHSN Location Groupers Selected, AU Data section

**Figure:**
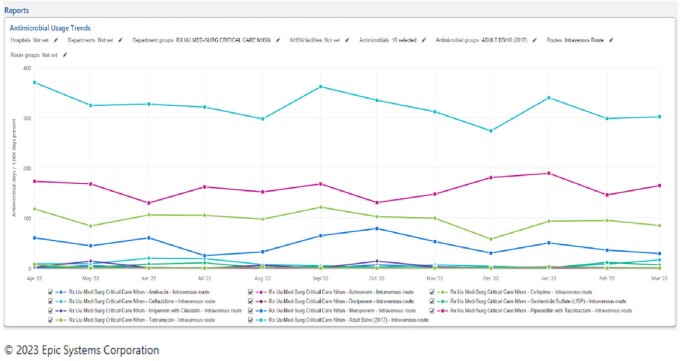

Pictured is the DOT for BSHO antimicrobials. The only input to see data was selecting the "department". The remaining information is prepopulated to the antimicrobial dashboard selected.

Optimized Dashboard With Units and Antimicrobial Grouper Selected, AU Data section

**Figure:**
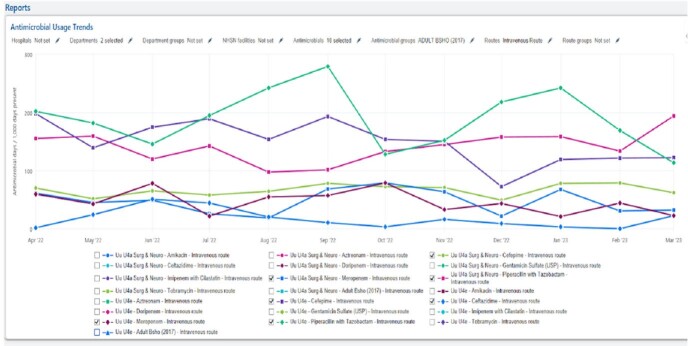

Pictured is same dashboard but manipulated to show the two units that make up the NHSN grouper. Additionally, antimicrobials with negligible DOT's have been deselected.

**Conclusion:**

We anticipate after the dashboards are fully implemented across the system, antibiotic stewardship site leads can integrate SAAR data into their stewardship program, giving SAAR a defined role in our health system's antimicrobial stewardship program management. As none of our configuration required changes to the module's source code, the EHR provider could have our optimized dashboards incorporated into the module's standard configuration. As more institutions receive SAAR reports, more stewardship programs will seek how to interpret and act on their SAAR data.

**Disclosures:**

**All Authors**: No reported disclosures

